# Factors associated with the Journal Impact Factor (JIF) for Urology and Nephrology Journals

**DOI:** 10.1590/S1677-5538.IBJU.2014.0497

**Published:** 2015

**Authors:** Joseph M. Sewell, Oluwakayode O. Adejoro, Joseph R. Fleck, Julian A. Wolfson, Badrinath R. Konety

**Affiliations:** 1 Department of Urology, University of Minnesota School of Medicine, Minneapolis, MN, USA;; 2 School of Public Health, Division of Biostatistics, University of Minnesota School of Medicine, Minneapolis, MN, USA

**Keywords:** Journal Impact Factor, Urology, Nephrology, Periodicals as Topic

## Abstract

**Purpose:**

The Journal Impact Factor (JIF) is an index used to compare a journal's quality among academic journals and it is commonly used as a proxy for journal quality. We sought to examine the JIF in order to elucidate the main predictors of the index while generating awareness among scientific community regarding need to modify the index calculation in the attempt to turn it more accurate.

**Materials and Methods:**

Under the Urology and Nephrology category in the Journal Citations Report Website, the top 17 Journals by JIF in 2011 were chosen for the study. All manuscripts’ abstracts published from 2009-2010 were reviewed; each article was categorized based on its research design (Retrospective, Review, etc). T and correlation tests were performed for categorical and continuous variables respectively. The JIF was the dependent variable. All variables were then included in a multivariate model.

**Results:**

23,012 articles from seventeen journals were evaluated with a median of 1,048 (range=78-6,342) articles per journal. Journals with a society affiliation were associated with a higher JIF (p=0.05). Self-citations (rho=0.57, p=0.02), citations for citable articles (rho=0.73, p=0.001), citations to non-citable articles (rho=0.65, p=0.0046), and retrospective studies (rho=-0.51, p=0.03) showed a strong correlation. Slight modifications to include the non-citable articles in the denominator yield drastic changes in the JIF and the ranking of the journals.

**Conclusion:**

The JIF appears to be closely associated with the number of citable articles published. A change in the formula for calculating JIF to include all types of published articles in the denominator would result in a more accurate representation.

## INTRODUCTION

**Figure 1:**
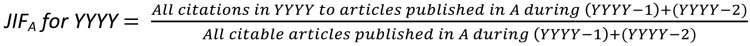
JIF Calculation formula.

The Journal Impact Factor (JIF) is an index annually published by The Journal Citation Reports (JCR). It was established by Eugene Garfield to compare investigators’ and journals’ research influence on its time (1). The JIF for a particular journal is calculated by the ratio of total number of citations received in a determined year to the total number of citable articles published by that journal in the previous 2 years (Figure-1) (2, 3). The non-citable articles criteria include editorials, news, meeting reports, etc. Original research and review articles are the only article types that meet the definition of citable articles according to the Institute of Scientific Information (3, 4). Web of Science (Thomson Reuters Inc.) is a citation service accessible through an indexing database and search engine, ISI Web of Knowledge^SM^ (WoK) (5–9).

The numerator of the JIF formula includes the citations of all articles of a given journal whereas its denominator excludes the number of non-citable articles published; thus making the formula potentially manageable. While this flaw has been regarded as insignificant by the index creator, it has also generated significant debate and skepticism among editors and authors who disagree. The creator himself has accepted the issue yet considering it “statistically significant only in rare cases” (2, 10–14). The exclusion of non-citable articles from the denominator can hypothetically improve the JIF by increasing the number of self-citations, non-citable articles or reducing the number of citable articles (15–17). Regardless of this situation, the JIF is held as the gold standard measure to judge journals’ quality with a general lack of awareness of its formula, purpose, and meaning. All of which is now coming under greater scrutiny (18–21, 14). This debate is not merely academic as many universities are now using “publishing in high JIF journals” as an evaluation criterion for promotion. Publication in high JIF journals also affects success in competing for extramural grant funding (22).

We sought to examine the JIF among nephrology and urology journals in order to elucidate the main predictors of the index while generating awareness among scientific community about the current formula used to calculate the JIF and its flaws. We also attempt to show that small discrepancies in the formula do influence the overall calculation and the subsequent ranking list.

## MATERIALS AND METHODS

### 

#### Journal Selection

The top 40 journals ranked by JIF in the Journal Citation Reports for 2011 in the urology and nephrology category were selected for the study. We excluded journals that were not published in English and published articles of one specific kind of research design (basic science, retrospective studies, review articles). Twenty-three journals out of the 40 journals initially selected were excluded for containing only review (16) and basic research (7) publications.

#### Variables Selection

All of the archived abstracts from each journal's website for years 2009-2010 were individually reviewed. This is the period used for JIF calculation that is reported for 2011. We categorized each of the articles according to research design (basic science, non-clinical experimental and translational research, clinical trial, retrospective study, prospective study, case report and case series, cross-sectional study, review article, meta-analysis, systematic review, and guideline). If the study design could not be ascertained from the abstract, the manuscript's methods section was read in order to obtain a more precise classification. Appendix 1 outlines the articles’ sorting criteria for research design (Appendix 1). The number of self-citations, citable and non-citable articles, and citations made to citable articles, and citations made to non-citable articles were also identified (15, 16). Other variables examined included society affiliation, US vs. non-US journals, number of articles published by the journal, average pages per issue, and US and non-US articles.

#### ISI Web of Knowledge^SM^ Citations Data Validation

Data values for JIF, self-citations, citable and non-citable articles published during 2009 and 2010, as well as the total number of citations used to calculate the 2011 JIF were retrieved and examined from the JCR web site (7, 8). The JCR website, however, did not individually disclose the citations made to citable and non-citable articles that were used to calculate the 2011 JIF. As an alternative, two other databases (Web of Science and MEDLINE) from the WoK website were used (5, 6, 9). The data for US and non-US articles was also acquired through the WoK website (6, 9).

Because the sum of citations to citable and non-citable articles (total number of citations) retrieved from the WoK website did not directly match the total number of citations acquired from the JCR website, we sought to validate the WOK website data by generating an alternative JIF utilizing the data extracted from the WOK website. The goal for this intermediate step was to accurately use the WOK website data a posteriori during our statistical analysis in lieu of the JCR website data (not individually disclosed).

The alternative JIF was calculated using the citations data acquired from the WoK website. This JIF was correlated with the official JIF published by JCR through the Spearman's rank-order correlation test. A correlation with a p-value less than 0.05 and a slope close to 1 were defined as the required criteria for validation.

### Statistical Analysis

The number of articles published during 2009 and 2010 issues published per year, average pages per issue, self-citations, issues published per year, average articles published per issue, citations made to citable articles, citations made to non-citable articles, and the categorical variables were analyzed as independent variables. All other continuous variables were analyzed as a ratio relative to the total number of publications in the period of interest$\frac{\text{number of review articles}}{\left(\text{i.e. total number of publications made during}2009\&201\right)}$because they depended on the total number of articles published.

The association between the variables and JIF was examined using the JIF as the dependent variable. A two tailed Welch's t-test and Spearman's rank-order correlation test were performed for categorical and continuous variables respectively. All variables were then included in a multivariate linear regression model using a stepwise variable selection method. An alternative multivariate model excluding the counts for citable articles was analyzed in order to account for co-linearity of other variables with the variable “citable articles”.

A hypothetical JIF (JIF’) was created with the data obtained from the JCR website, one that includes the non-citable articles in the denominator of the index calculation (Citations made to Citable and Non-citable articles/Citable and Non-citable articles). A Welch's t-test was also used to compare the JIF published by the JCR with the hypothetically created JIF. All computations were performed using SAS 9.3 (SAS Institute, Cary, NC). P-values of <0.05 were considered to be statistically significant. All the assumptions were verified for every test.

## RESULTS

A total of 12/17 journals were society affiliated, 8 were US originated, and 9 were non-US originated. Table-1 summarizes all the variables used in the analysis (Table-1). A total of 23,012 article abstracts were examined with a median of 1,048 (range=78–6,342) articles per journal.

**Table 1:** Journals' summary statistics and Analyses Results.

Dependent Variable	Total Number	Median (Range)	–	–
Journal Impact Factor	17	3.22 (2.10-9.66)	–	–
Categorical Variables	Total Number	JIF Mean (CI-95%)	*t*-value (df) −0.18 (15)	*p*-value
US Journals	9	4.08 (2.01-6.16)	0.86 (15)	0.86
Non-US Journals	8	3.88 (2.20-5.55)	–	–
Society Affiliated Journals	12	4.46 (2.86-6.07)	-2.14 (13)	0.05*
Non-society Affiliated Journals	5	2.80 (2.06-3.54)	–	–
Continuous Variables	Total Number	Median (Range)	Rho (*R* ^2^)	*p*-value
*Average Pages Per Issue	179.3	154.9 (31.54-424.58)	0.36 (0.13)	0.16
Total Number of Publications	23012	1048 (78-6342)	0.30 (0.09)	0.25
US Articles	7961	357 (20-1712)	0.46 (0.22)	0.06
Non-US Articles	15051	535 (58-4630)	-0.46 (0.22)	0.06
Citable Articles	9598	488 (31-1437)	-0.29 (0.08)	0.26
Citations to Citable Articles	36859	2497 (58-4630)	0.73 (0.53)	0.001*
Non-citable Articles	13595	467 (2-1437)	0.29 (0.08)	0.26
Citations to Non-citable Articles	1790	87 (0-234)	0.65 (0.42)	0.0046*
Journal Self-Citations	5255	240 (0-1339)	0.57 (0.32)	0.02*
Retrospective	2616	54 (14-613)	-0.51 (0.26)	0.03*
Clinical Trials	621	18 (6-108)	-0.01 (0.00)	0.96
Basic Science Research	2776	128 (5-287)	-0.05 (0.00)	0.84
Prospective	1659	64 (8-337)	-0.44 (0.20)	0.07
Case Report/Case Series	683	9 (2-199)	-0.47 (0.22)	0.05*
Cross-Sectional	313	7 (1-127)	-0.05 (0.00)	0.83
Review Articles	824	42 (2-117)	-0.03 (0.00)	0.89
Meta-analysis	53	1 (0-13)	0.03 (0.00)	0.92
Systematic Review	41	1 (0-10)	-0.18 (0.03)	0.50
Guidelines	45	0 (0-25)	0.23 (0.05)	0.37

### 

#### ISI Web of Knowledge^SM^ Citations Data Validation

**Figure 2:**
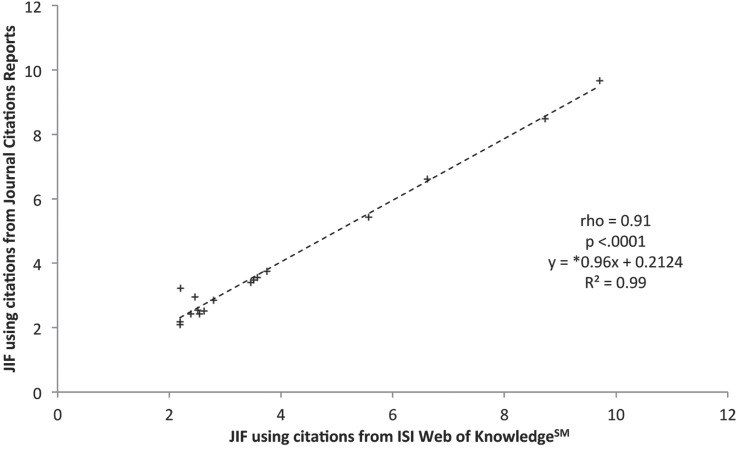
The scattered plot exhibits the correlation between Journal Impact factors calculated using data retrieved from web of Knowledge, and data retrieved from Journal Citation Reports. *slope (m).

While utilizing the WOK website data 11 journals increased their JIF, 5 decreased their JIF, and 1 was unaffected. The Journal of Urologic Oncology: Seminars and Original Investigations, and The Journal Neurourology and Urodynamics had an important citations count miss-match between databases, which resulted in a JIF decrement of 0.5 points or more. The JIF calculated using the WoK website data correlated well with the JIF calculated using the JCR website data (rho=0.91, p<0.001, m=0.96) (Figure-2). Once validated, the data gathered from the WoK website was used to calculate a hypothetical JIF excluding citations accounted to non-citable articles (Table-2). This exclusion represented a consistent drop in the JIF value throughout the 17 journals, promoting at the same time ranking position variations.

**Table 2:** Data Extracted from Journal Citations Reports and web of Knowledge^sM^

	JIF-JCR	JIF-WOK*	JIF'	NCA	CA	Total Cites-WOK^Δ^	Total Cites-JCR
J Am Soc Nephrol	9,66	9,71	7,49	143	493	4785	4764
Eur Urol	8,49	8,73	3,70	592	456	3982	3876
Kidney Int	6,61	6,62	3,63	475	578	3825	3818
Am J Kidney Dis	5,43	5,57	2,02	824	488	2718	2652
J Urology	3,75	3,75	0,85	4905	1437	5383	5383
J Sex Med	3,55	3,58	1,24	1413	756	2707	2685
Prostate	3,49	3,51	3,45	4	361	1267	1258
Nephrol Dial Transplant	3,40	3,46	2,77	272	1193	4126	4051
Urol Oncol-Semin Ori	3,22	2,20	2,61	46	199	438	640
Neurourol Urodynam	2,96	2,46	0,61	1016	262	645	775
Bju Int	2,84	2,80	1,44	988	1016	2840	2890
Am J Nephrol	2,54	2,51	2,35	23	282	708	716
Pediatr Nephrol	2,52	2,62	0,58	1815	548	1437	1380
Urology	2,43	2,54	1,32	956	1140	2900	2768
Prostate Cancer P D	2,42	2,39	2,21	11	114	272	276
Bmc Nephrol	2,18	2,19	2,18	0	68	149	148
Periton Dialysis Int	2,10	2,19	1,36	112	207	467	434

JIF-JCR mean = 3.97 vs. JIF' mean = 2.34, *t*-Test (29) = 2.41, *p*-value= 0.02

JIF: Journal Impact Factor, WOK: Web of Knowledge^SM^, CA: Citable Articles, NCA: Non-Citable Articles

* JIF using data extracted from Web of Knowledge.

JIF' calculated including Non-citable articles in the denominator; Formula: JIF=Total Cites-JCR/(NCA+CA)

^Δ^Calculated by adding up Citations to CA and NCA.

### Statistical Analysis

Journals with a society affiliation were associated with a higher JIF compared to journals without a society affiliation (mean JIF=4.46 vs. 2.80, p=0.05). US journal origin vs. non-US journal origin did not demonstrate an association with JIF (mean JIF=4.08 vs. 3.88, p=0.86) (Table-1). A significant difference was found while comparing the JIF published by the JCR with the hypothetical JIF including the non-citable articles in the denominator of the calculation (mean JIF=3.97 vs. mean JIF'=2.34, p=0.02) (Table-2).

The bivariate analysis also found some of the examined variables to be significantly associated with JIF: self-citations (rho=0.57, p=0.02), retrospective studies (rho=-0.51, p=0.03), citations to citable articles (rho=0.73, p=0.001), citations to non-citable articles (rho=0.65, p=0.0046), and case reports/case series (rho=-0.47, p=0.05) (Table-1). The remaining variables examined were not found to be good predictors of JIF.

The multivariate linear regression analysis demonstrated that citations made to citable articles (p<0.001) are the most important predictor of a high JIF, whereas the number of citable articles (p=0.015), total number of articles published (p<0.001) and case reports/case series (p=0.002) are the most important predictors of low JIF (Table-3). Appendix 2 summarizes the multivariate model excluding the variable citable articles.

**Table 3:** Results for the Multiple Linear Regression Model.

Multivariate Model	Estimate	Standard Error	Adjusted Estimate (*t*)*	*p*-value
Intercept	5.13	0.87	5.9	–
Total Articles published	-0.0014	0.00022	-6.36	<0.001
Citable Articles	-2.98	1.045	-2.85	0.015
Case Reports/Case Series	-28.03	7.3	-3.84	0.002
Citations to Citable Articles	0.0015	0.00016	9.38	<0.001

* Adjusted estimate by variable scale (normalized estimate: Estimate/Standard Error). **Model R^2^ = 0.9**

## DISCUSSION

The findings of this study emphasize the need to modify the JIF formula in order to make it more accurate and acceptable. No prior publications have focused on this topic in Urology, nor has a detailed statistical analysis been performed to weight the different variables that are included in the JIF. Variables such as citations to citable and non-citable articles, self-citations, and low number of citable articles published have been hypothesized as predictors of high JIF (15, 16, 17). These assumptions were held as true during this analysis and confirm them.

Regardless of having readily available counts of total number of citations the JCR website does not routinely disclose the individual counts for citations made to citable and non-citable articles, and such numbers can only be obtained indirectly through the WoK website (7, 8). The data acquired from the WoK and JCR websites were compared in order to assess the feasibility of the analysis. Regardless of being under the same umbrella, differences between the two datasets were found. Due to these discrepancies, we sought to validate the citations data retrieved from the WoK website against the data published in the JCR website. With the proper computing, a strong validation was indeed achieved with excellent similarity between the two data-sets (rho=0.91, slope=0.96) (Figure-2).

The validation process enabled us to prove that citations to non-citable articles do signifcantly improve the JIF (rho=0.65, p=0.0046) (15, 16). Also, a high proportion of retrospective studies and case reports/case series had a negative effect over the JIF. With few exceptions retrospective studies and case reports/case series are regarded as a resource of low evidence level, hence tend to have a low impact in the scientific community and do not receive as many citations as designs regarded as higher level of evidence (i.e. meta-analyses) (23). Therefore the inclusion of non-citable articles and exclusion of case reports/case series and retrospective studies may serve as positive predictors of JIF. Low citable article counts and high self-citation rates also appear to predict the JIF (Table-1). The latter echoes the result of a previous analysis including 6 anesthesiology journals (17).

Citations made to citable articles were the most important variable influencing the JIF. Articles reporting the results of large clinical trials, basic science research, cross-sectional studies, reviews, systematic reviews, and meta-analysis did not demonstrate a significant correlation with the JIF nor were they independent predictors of JIF when controlling for all other factors. This could be due to the fact that research design variables are included under the umbrella of citable articles making them collinear variables. However, a sensitivity analysis was performed in order to control for co-linearity among the research design variables by eliminating the citable articles variable. The results were not modified (Appendix 2).

Because citable articles are fundamental for the JIF calculation, such model was performed only to account for co-linearity and does not provide any other useful insight.

The journals with highest JIF in our cohort grossly were those that accepted the least number of retrospective studies and case reports/case series. The concern with this fact is that journal editors could place greater emphasis on the “citability” of an article rather than the quality and nature of the scientific question. Much of the literature in Urology focuses on certain subspecialties such as oncology, which makes these articles potentially more citable and getting higher priority for publication. The citability of an article could also be linked to the topical nature of the article or in other words, its conformation to the existing trend. In example, multiple articles on certain popular topics such as robotic surgery could be published and crowd out excellent work in basic science and less popular areas.

One other interesting finding was that the JIF of journals linked to professional societies had a higher value. This may represent the fact that members of the society are more likely to be aware of and cite articles from the journal of their society. This may be an unrecognized value to the journals and to the societies since publishing in higher JIF journals is becoming increasingly important to academicians and increasing JIF is important to journals.

When a hypothetical JIF including the non-citable articles in the denominator was created, a significant difference was found when compared to the JIF published by the JCR (p=0.02). This analysis contrasts with the belief of finding “significant discrepancies only in rare cases” (2). Moreover, this hypothetical modification of the JIF generated multiple rank changes among the journals. This issue builds a compelling case regarding the need to modify the formula to calculate the JIF since it is regarded as gold standard of journal quality and often looked at as a criterion for promotion or extramural grant funding (Table-2) (18-22).

The limitations of this study include the fact that we examined the correlation of articles to JIF over a two-year snapshot in time. Factors involved and associations could change over time and these data do not have the ability to capture that. We identified and analyzed a set of journals in a non-randomized manner. Hence we are making the assumption that these findings hold true for journals that are not the top 40 in the Urology/Nephrology category. These findings also apply only to English language journals. The biggest limiting factor lies on the lack of access to citations counts for each research design, which is essential to assess the influence of research designs over the JIF prediction. Despite these shortcomings, this is the first study to perform a detailed analysis of JIF among urology journals. These data can provide us with some understanding of what predicts JIF.

## CONCLUSIONS

Variations to the formula to account for non-citable articles in the denominator do represent a statistical significant difference when compared to the JIF as it is currently calculated. A change in the formula for calculating JIF to include all types of published articles in the denominator would result in a more accurate and accepted representation. The JIF appears to be predicted by the number of citations made to citable articles and non-citable articles, self-citations, case reports/case series, retrospective studies and citable articles published. Number of citations made to each different research design is necessary in order to assess their influence on the JIF.[Table t4] [Table t5]

## Appendix 1 Articles’ Sorting Criterion According to Research Design.

[Table t4]

**Table t4:**

Retrospective studies	Retrospective designs, including retrospective center and professional experience; retrospective and prospectively generated databases.
Clinical Trials	Experimental clinical studies such as randomized and n on -randomized, blinded and non-blinded clinical trials.
Basic science research	Basic science research, animal experimental and observational research, non-clinical meta-analysis, and translational research.
Prospective studies	Prospective observational studies, including longitudinal studies, and short follow up cohort observational studies. No databases v/here included in these group as they belong to retrospective studies.
Case Report/Case Series	Case reports, case series, description of novel procedures and merely descriptive articles.
Cross-sectional studies	Cross-sectional studies. By definition, data collection takes place in over a fixed amount of time any follow up. Data retrospectively acquired defaulted the abstract to be classified as a retrospective study.
Review articles	Review articles without any fixed methodology or criteria for revision.
Meta-analysis	Meta-analyses, systematic review and meta-analyses.
Systematic reviews	Systematic reviews without meta-analyses.
Guidelines	Guidelines, professional consensus and recommendations, reference material, expert opinion, and best practices.
N on -citable articles	Editorials, letters, replies, commentaries, erratum, diaries, debates, summaries, news, biographical articles, meeting abstracts, meeting podiums. meeting posters, points and counterpoints and other journal specific categories.

## Appendix 2 Results for the Multiple Linear Regression Model Excluding Citable Articles.

[Table t5]

**Table t5:**

Multivariate Model	Estimate	Standard Error	Adjusted Estimate (Estimate/SE)	*p*–value
Intercept	2.28	0.46	4.96	–
Total Publications	-0.001	0.00022	-4.55	<0.001
Non-Citable Articles	2.75	1.032	2.66	0.02
Case Reports/Case Series	-28.95	7.4	-3.91	0.002
Citations to Citable Articles	0.00147	0.00016	9.19	<0.001

Model R^2^ = 0.9
